# A real-world study of anlotinib as third-line or above therapy in patients with her-2 negative metastatic breast cancer

**DOI:** 10.3389/fonc.2022.939343

**Published:** 2022-07-28

**Authors:** Yingbo Shao, Zhifen Luo, Yang Yu, Yaning He, Chaojun Liu, Qi Chen, Fangyuan Zhu, Bing Nie, Hui Liu

**Affiliations:** ^1^ Department of Breast Oncology, Henan Provincial People’s Hospital; Zhengzhou University People’s Hospital, Zhengzhou, China; ^2^ Department of Breast Oncology, Henan Provincial People’s Hospital; Henan University People’s Hospital, Zhengzhou, China; ^3^ Department of Medical Oncology, Henan Provincial People’s Hospital; Zhengzhou University People’s Hospital, Zhengzhou, China; ^4^ Department of Medical Oncology, Henan Provincial People’s Hospital; Henan University People’s Hospital, Zhengzhou, China

**Keywords:** breast cancer, anlotinib, anti-angiogenesis, immunotherapy, safety

## Abstract

**Background:**

Antiangiogenic agents provides an optional treatment strategy for patients with metastatic breast cancer. The present study was conducted to evaluate the efficacy and safety of anlotinib as third-line or above therapy for patients with HER-2 negative metastatic breast cancer.

**Methods:**

Patients with HER-2 negative metastatic breast cancer who have failed from prior therapy and treated with anlotinib monotherapy or combined with chemotherapy or immunotherapy from June 2018 to December 2020 were retrospectively analyzed based on real-world clinical practice. The primary end point was progression free survival (PFS). Secondary end points included objective response rate (ORR), disease control rate (DCR), overall survival (OS) and safety.

**Results:**

47 patients with HER-2 negative metastatic breast cancer received anlotinib monotherapy or combination therapy as third-line or above therapy. In the general population, 10 patients achieved PR, 25 patients had SD and 12 patients had PD. The overall ORR and DCR were 21.3% and 74.5%, respectively. Subgroup analysis suggested that there were no statistically significant differences in ORR and DCR with respect to HR status (positive vs. negative), treatment programs (monotherapy vs. combination) and treatment type in combination group (chemotherapy vs. immunotherapy). The patients who did not received previously anti-angiogenesis therapy had superior DCR (84.8% vs. 50.0%, P=0.012). Median PFS and OS were 5.0 months (95% CI=4.3-5.7) and 21.0 (95% CI=14.9-27.1) months, respectively. The PFS (6.5m vs. 3.5m, P=0.042)and OS (28.2m vs. 12.6m, P=0.040) were better in HR positive patients than HR negative patients. And simultaneously, patients who received anlotinib combination therapy obtained better PFS (5.5m vs. 3.0m, P=0.045). The incidence of Grade 3-4 adverse events(AEs) was 31.9%.

**Conclusions:**

Anlotinib monotherapy or combination therapy provide a viable third-line or above therapeutic strategy in patients with HER-2 negative metastatic breast cancer, a median PFS of 5.0 months was obtained with well tolerated toxicity.

## Introduction

According to the global cancer registration data in 2020, breast cancer has become the most common malignant tumor in the world, accounting for 11.7% of all cancers (1). Through the development of medicine in the past decade, the treatment level of breast cancer has undergone tremendous changes. The improvement of the overall treatment level of breast cancer benefits from the deepening understanding of the biological behavior of breast cancer and the progress of comprehensive treatment methods, including chemotherapy, targeted therapy, endocrine therapy and immunotherapy (2–5). Even after comprehensive treatment, a considerable proportion of patients will progress to metastatic breast cancer, which still lacking effective treatment manner.

Angiogenesis is one of the key factors of tumorigenesis and progression, and it is also a significant feature of malignant tumors. Anti-angiogenesis is considered to be an important therapeutic strategy for the treatment of various tumors, especially for advanced or metastatic cancer (6). Antiangiogenic drugs have shown good efficacy in a variety of solid tumors, including lung cancer, gastric cancer, colorectal cancer, gynecological tumors and so on (7–10). However, the value of antiangiogenic drugs in metastatic breast cancer is still controversial, including bevacizumab, ramucirumab, and et al (11–13). Bevacizumab is the first antiangiogenic drug used in metastatic breast cancer. Clinical studies have shown that bevacizumab can prolong PFS, but cannot improve OS, and may be accompanied by serious adverse reactions (14–16).

In recent years, small-molecule multi-targeted tyrosine kinase inhibitors (TKIs) have shown good antitumor activity and have become a new therapeutic strategy for many malignant tumors. Previous phase II clinical studies have confirmed the efficacy and safety of apatinib monotherapy in patients with metastatic triple-negative breast cancer (17). As a novel oral TKI, which can effectively inhibit vascular endothelial growth factor receptor (VEGFR), platelet-derived growth factor (PDGFR), fibroblast growth factor receptor (FGFR) and c-Kit, anlotinib has proven efficacy in many solid tumors (18–20). The present study was conducted to evaluate the efficacy and safety of anlotinib as third-line or above therapy for patients with HER-2 negative metastatic breast cancer based on real-world clinical practice.

## Patients and methods

### Patients population

From June 2018 to December 2020, patients with HER-2 negative metastatic breast cancer who received third-line or above therapy in Henan Provincial People’s Hospital were screened, and patients with HER-2 negative metastatic breast cancer who received anlotinib as third-line or above therapy were enrolled and analyzed for efficacy and safety. The main selection criteria included: 1) histopathological confirmed metastatic breast cancer; 2) HER-2 negative by immunohistochemistry or fluorescence *in situ* hybridization (FISH); 3) have received at least second-line system rescue treatment; 4) received anlotinib as third-line or above therapy; 5) at least one measurable lesion based on RECIST v1.1. The main exclusion criteria included: 1) received less than two cycles of anlotinib treatment and could not evaluate the efficacy; 2) efficacy evaluation and follow-up data are not available.

### Study treatment

In this study, the patients received anlotinib monotherapy or combination therapy until disease progression, unacceptable toxicity or death. In the monotherapy regimen, anlotinib was given at a dose of 12mg as the initial dose once a day on d1 to d14 every three weeks. In the combination therapy regimen, anlotinib was given at a dose of 10 mg once a day on d1 to d14 every three weeks, and chemotherapy or immunotherapy were given simultaneously. Regarding chemotherapy, the regimen includes capecitabine and albumin bound paclitaxel. Capecitabine was provided as tablets and administered orally at a dose of 1000 mg/m^2^, bid, d1-d14, q21d. Albumin bound paclitaxel was administered intravenously at a dose of 100 mg/m^2^ on d1, q7d. With respect to immunotherapy, pembrolizumab was administered intravenously at a dose of 200 mg once every three weeks.

### Efficacy and safety assessments

After treatment, imaging examinations were performed after every two cycles of treatment in all patients to evaluate the clinical efficacy. The efficacy evaluation criteria are RECIST version 1.1 response evaluation criteria in solid tumors. According to RECIST version 1.1 response evaluation criteria, the efficacy assessment is divided into complete response (CR), partial response (PR), stable disease (SD), and progressive disease (PD). The objective response rate (ORR) was defined as CR + PR, and the disease control rate (DCR) was CR+ PR and SD. The toxicity was assessed according to the Common Terminology Criteria for Adverse Events, version 4.0.

### Statistical analysis

All the statistical analyses were performed with SPSS 22.0 software (SPSS Inc., IL, US) software. Survival curves of patients were estimated by the Kaplan-Meier method and compared using the log-rank test. The follow-up deadline is March 15, 2022. Progression-free survival (PFS) was defined as starting anlotinib monotherapy or combination therapy as third-line or above treatment to disease progression or death. Overall survival (OS) was defined as the period from the time of anlotinib monotherapy or combination therapy as third-line or above therapy to patient death or last follow-up. Difference between groups were determined by Pearson’s chi squared test or Fisher’s exact test. P<0.05 was considered significant.

## Results

### Patient and treatment characteristics

A total of 47 patients with HER-2 negative metastatic breast cancer who received anlotinib as third-line or above therapy were included. Patient and treatment characteristics are summarized in **Table 1**. The median age was 51 years (range 25-70), with 46 female patients and 1 male patients. In total, 25 (53.2%) patients were hormone receptor positive/HER2-negative, and 22 (46.8%) patients were TNBC. Most patients (43, 91.5%) were ECOG PS 0-1, and the other 4 (8.5%) patients were ECOG PS 2. All patients were diagnosed as recurrent and metastatic breast cancer. The common metastatic sites included lymph node (57.4%), chest wall (21.3%), liver (36.2%), lung (61.7%), bone (42.6%) and brain (31.9%). Number of metastatic sites in 18 (38.3%) patients were 1 or 2, and the other 29 (61.7%) patients were 3 or more. Thirty-eight patients (80.9%) had visceral metastasis, and the other 9 patients (19.1) had non-visceral metastasis. Six patients were in advanced stage at the time of initial diagnosis, and the other 41 patients were diagnosed as recurrent breast cancer. All the 41 patients had undergone previous breast surgery, and forty of these patients had received adjuvant therapy.

**Table 1:** Patient and treatment characteristics.

Characteristic	Total (n=47) n (%)	HR positive(n=25) n (%)	HR negative(n=22) n (%)
**Age (years, median)**	51 (25-70)	52 (31-70)	51 (25-69)
**Gender**			
Female	46 (97.9)	24 (96.0)	22 (100)
Male	1 (2.1)	1 (4.0)	0 (0)
**ECOG**			
0-1	43 (91.5)	23 (92.0)	20 (90.9)
2	4 (8.5)	2 (8.0)	2 (9.1)
**Metastatic type**			
Locoregional	4 (8.5)	0 (0)	4 (18.2)
Distant	43 (91.5)	25 (100)	18 (81.8)
**Metastatic site**			
Lymph node	27 (57.4)	14 (56.0)	13 (59.1)
Chest wall	10 (21.3)	4 (16.0)	6 (27.3)
Liver	17 (36.2)	9 (36.0)	8 (36.4)
Lung	29 (61.7)	19 (76.0)	10 (45.5)
Bone	20 (42.6)	11 (44.0)	9 (40.9)
Brain	15 (31.9)	7 (28.0)	8 (36.4)
Others	13 (27.7)	9 (36.0)	4 (18.2)
**Number of metastatic sites**			
1-2	18 (38.3)	5 (20.0)	13 (59.1)
≥ 3	29 (61.7)	20 (80.0)	9 (40.9)
**Metastatic sites type**			
Visceral	38 (80.9)	21 (84.0)	17 (77.3)
Non-Visceral	9 (19.1)	4 (16.0)	5 (22.7)
**Prior therapies before metastasis**			
Surgery	41 (87.2)	21 (84.0)	20 (90.9)
Neoadjuvant	6 (12.8)	5 (20.0)	1 (4.5)
Adjuvant	40 (85.1)	21 (84.0)	19 (86.4)
**Prior chemotherapy after metastasis**			
Taxanes	35 (74.5)	18 (72.0)	17 (77.3)
X/N/G	41 (87.2)	20 (80.0)	21 (95.5)
Platinum	29 (61.7)	15 (60.0)	14 (63.6)
Others	8 (17.0)	3 (12.0)	5 (22.7)
**Prior endocrine therapy after metastasis**			
AI	19 (40.4)	19 (76.0)	0 (0)
Fulvestrant	10 (21.3)	10 (40.0)	0 (0)
CDK4/6+	4 (8.5)	4 (16.0)	0 (0)
**Prior anti-angiogenesis after metastasis**			
Yes	14 (29.8)	6 (24.0)	8 (36.4)
No	33 (70.2)	19 (76.0)	14 (63.6)
**Treatment programs**			
Monotherapy	14 (29.8)	8 (32.0)	6 (27.3)
Combination	33 (70.2)	17 (68.0)	16 (72.7)
**Treatment line**			
3	19 (40.4)	10 (40.0)	9 (40.9)
≥ 4	28 (59.6)	15 (60.0)	13 (59.1)

HR, hormone receptor; ECOG, Eastern Cooperative Oncology Group; X, capecitabine; N, vinorelbine; G, gemcitabine; AI, aromatase inhibitor; CDK4/6, cyclin-dependent kinase 4 and 6.

During rescue therapy stage, 74.5% of patients had received taxanes treatment, 87.2% had received X/N/G treatment, including capecitabine, vinorelbine and gemcitabine, and 61.7% had received platinum treatment. For HR positive breast cancer patients, 76.0% of patients had received AI endocrine therapy, 40.0% had received fulvestrant endocrine therapy, and only 4 patients had received cyclin-dependent kinase 4 and 6 (CDK4/6) inhibitor treatment. In the general population, 14 (29.8%) patients had received anti-angiogenesis therapy, including bevacizumab or apatinib. All the patients included in this study had received at least two lines of rescue therapy, so anlotinib was given as third-line in 19 (40.4%) patients, and as forth-line or above therapy in 28 (59.6%) patients. 14 (29.8%) patients received anlotinib monotherapy, and the other 33 (70.2%) patients received anlotinib combination therapy. In the combination group, chemotherapy and immunotherapy were used in 26 and 7 patients, respectively.

### Efficacy

In the general population, CR was not observed, 10 patients achieved PR, 25 patients had SD and 12 patients had PD. The overall ORR and DCR were 21.3% (10/47) and 74.5% (35/47), respectively (**Table 2**). In HR positive breast cancer patients, CR was not observed, 5 patients achieved PR, 16 patients had SD and 4 patients had PD. The overall ORR and DCR were 20.0% (5/25) and 84.0% (21/25), respectively. In HR negative breast cancer patients, CR was not observed, 5 patients achieved PR, 9 patients had SD and 8 patients had PD. The overall ORR and DCR were 22.7% (5/22) and 63.6% (14/22), respectively. The ORR in anlotinib monotherapy and combination therapy group were 21.4% and 21.2%, respectively. The DCR in anlotinib monotherapy and combination therapy group were 64.3% and 78.8%, respectively. In anlotinib combination therapy group, the ORR and DCR in anlotinib plus chemotherapy group were 19.2% and 76.9%, and 28.6% and 85.7% in anlotinib plus immunotherapy group, respectively. There were no statistically significant differences in ORR and DCR with respect to HR status (positive vs. negative), treatment programs (monotherapy vs. combination) treatment type in combination group (chemotherapy vs. immunotherapy), metastatic sites type (visceral vs. non-visceral), number of metastatic sites (1-2 vs. ≥ 3) and prior chemotherapy after metastasis (with taxanes vs. without taxanes, **Supplementary Table 1**). The patients who did not received prior anti-angiogenesis therapy had higher DCR than patients who had received prior anti-angiogenesis therapy (84.8% vs. 50.0%, P=0.012), but there was no statistical difference in ORR between the two groups (24.2% vs. 14.3%, P=0.446, **Table 2**). There were no significant differences in ORR and DCR among different chemotherapy and immunotherapy medications in the combination group (**Supplementary Table 1**).

**Table 2:** Efficacy of anlotinib treatment in patients with Her-2 negative metastatic breast cancer.

Parameter	Best response				ORR	*P*	DCR	*P*	Median PFS (95%CI)	*P*	Median OS (95%CI)	*P*
	CR	PR	SD	PD								
**Total**	0	10	25	12	21.3 (10/47)	–	74.5 (35/47)	–	5.0 (4.3-5.7)	–	21.0 (14.9-27.1)	–
**HR status**						0.820		0.110		**0.042**		**0.040**
HR positive	0	5	16	4	20.0 (5/25)		84.0 (21/25)		6.5 (4.7-8.3)		28.2 (18.8-37.6)	
HR negative	0	5	9	8	22.7 (5/22)		63.6 (14/22)		3.5 (2.2-4.8)		12.6 (4.4-20.8)	
**Treatment programs**						0.987		0.297		**0.045**		0.695
Monotherapy	0	3	6	5	21.4 (3/14)		64.3 (9/14)		3.0 (2.0-4.0)		27.5 (9.0-46.0)	
Combination	0	7	19	7	21.2 (7/33)		78.8 (26/33)		5.5 (4.3-6.7)		21.0 (13.8-28.2)	
**Treatment type in combination group**						0.623		1.000		0.797		0.293
Anlotinib + chemotherapy	0	5	15	6	19.2 (5/26)		76.9 (20/26)		5.0 (4.0-6.0)		15.7 (3.9-27.5)	
Anlotinib + immunotherapy	0	2	4	1	28.6 (2/7)		85.7 (6/7)		6.6 (2.5-10.7)		22.2 (NE)	
**Prior anti-angiogenesis therapy**						0.446		**0.012**		0.158		0.885
Yes	0	2	5	7	14.3 (2/14)		50.0 (7/14)		2.3 (0.3-4.3)		21.0 (7.4-34.6)	
No	0	8	20	5	24.2 (8/33)		84.8 (28/33)		5.5 (4.3-6.7)		21.5 (14.8-28.2)	

CR, complete response; PR, partial response; SD, stable disease; PD, progressive disease; ORR, overall response rate; DCR, disease control rate; PFS, progression free survival; OS, overall survival; *: Vs. chemotherapy. Bold values: P < 0.05.

**Figure 1:**
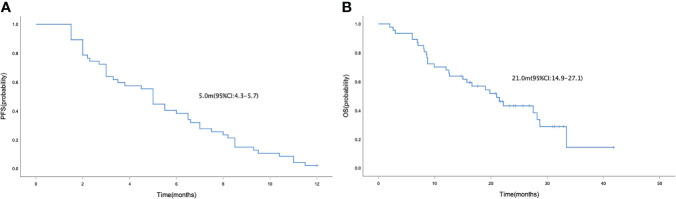
Kaplan-Meier curve of PFS **(A)** and OS **(B)** in the general population who received anlotinib as third-line or above therapy for patients with HER-2 negative metastatic breast cancer.

**Figure 2:**
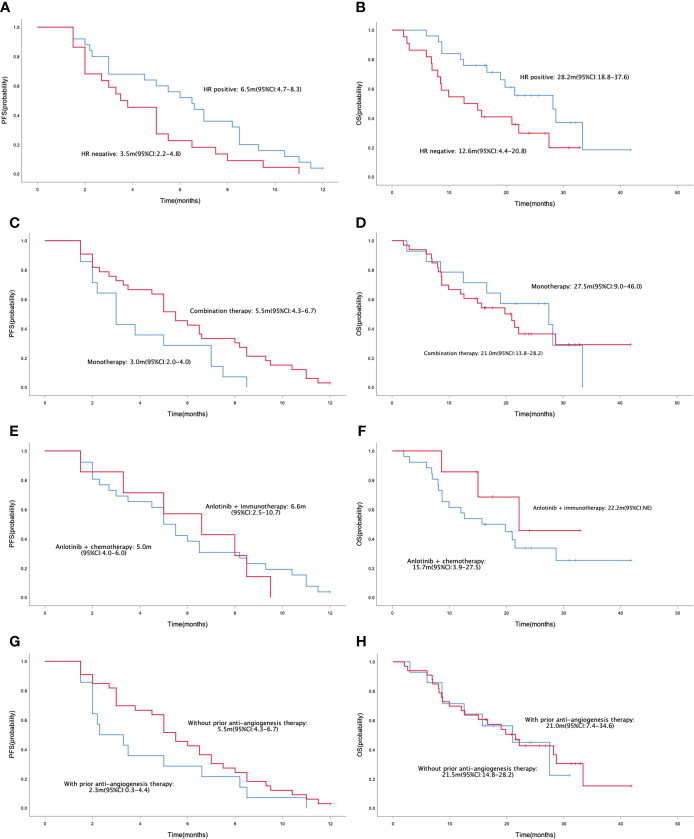
Kaplan-Meier curve of PFS **(A)** and OS **(B)** in patients with different HR status (HR positive vs. HR negative). Kaplan-Meier curve of PFS **(C)** and OS **(D)** in patients with different treatment programs (monotherapy vs. combination therapy). Kaplan-Meier curve of PFS **(E)** and OS **(F)** in patients with different treatment type in combination group (anlotinib + chemotherapy vs. anlotinib + immunotherapy). Kaplan-Meier curve of PFS **(G)** and OS **(H)** in patients with or without prior anti-angiogenesis therapy.

In the general population, the median PFS and median OS were 5.0 (95% CI= 4.3-5.7) and 21.0 (95% CI= 14.9-27.1) months, respectively (**Figures 1A, B**). The median PFS were 6.5 (95% CI= 4.7-8.3) and 3.5 (95% CI= 2.2-4.8) months in the HR positive and negative population, respectively (P = 0.042; **Figure 2A**). The median OS in the two groups were 28.2 (95% CI=18.8-37.6) months and 12.6 (95% CI=4.4-20.8) months, respectively(P = 0.040; **Figure 2B**). The PFS and OS were better in HR positive patients than HR negative patients. In the meantime, patients who received anlotinib combination therapy obtained better PFS than those who received anlotinib monotherapy (5.5m vs. 3.0m, P=0.045; **Figure 2C**). However, the OS was not statistically different between the two groups (21.0m vs. 27.5m, P=0.695; **Figure 2D**). There were no statistically significant differences in PFS and OS with respect to treatment type in combination group (plus chemotherapy vs. immunotherapy; **Figures 2E, F**) and prior anti-angiogenesis therapy (Yes vs. No; **Figures 2G, H**). There were also no significant differences in PFS and OS in metastatic sites type (visceral vs. non-visceral), and prior chemotherapy after metastasis (with taxanes vs. without taxanes). However, the median OS in patients with 1-2 metastatic sites was better than in those with ≥ 3 metastatic sites (**Supplementary Table 1**). And simultaneously, the median PFS in patients with 1-2 metastatic sites was longer, but no significant difference was found. No significant differences were found in treatment type in combination group among anlotinib plus capecitabine, nab-paclitaxel or pembrolizumab (**Supplementary Table 1**).

### Safety

Most of the adverse events in patients received anlotinib therapy were grade 1-2 in severity, and no unexpected side effects or treatment-related death occurred (**Table 3**). The incidence of Grade 3-4 AEs was 31.9%. In anlotinib monotherapy group, Non-hematological treatment-related AEs were secondary hypertension (n=6, 42.9%), hand-foot syndrome (n=5, 35.7%), proteinuria (n=2, 14.3%), fatigue (n=5, 35.7%), anorexia (n=2, 14.3%), diarrhea (n=2, 14.3%), rash (n=1, 7.1%), oral mucositis (n=2, 14.3%) and gum bleeding (n=1, 7.1%). Hematological AEs were decreased platelet, increased ALT/AST, dyslipidemia and TSH elevation. The incidence of adverse reactions was generally low. Grade 3-4 AEs were secondary hypertension (n=2,14.3%), hand-foot syndrome (n=1, 7.1%), and proteinuria (n=1,7.1%). The dose of anlotinib was reduced from 12 mg to 10 mg due to adverse reactions in 2 patients. The remaining patient could tolerate the treatment by suspending the medication.

**Table 3:** Treatment-Related AEs (TRAEs).

Adverse Event	Combination therapy				Monotherapy	
	All Grade	≥ Grade3			All Grade	≥ Grade3
**Non-hematologic**						
Secondary hypertension	15 (45.5)		2 (6.1)		6 (42.9)	2 (14.3)
Hand-foot syndrome	16 (48.5)		2 (6.1)		5 (35.7)	1 (7.1)
Proteinuria	6 (18.2)		0		2 (14.3)	1 (7.1)
Fatigue	13 (39.4)		0		5 (35.7)	0
Nausea or Vomiting	10 (30.3)		2 (6.1)		0	0
Anorexia	9 (27.3)		0		2 (14.3)	0
Diarrhea	10 (30.3)		1 (3.0)		2 (14.3)	0
Muscle pain/joint pain	5 (15.2)		0		0	0
Sensory neurotoxicity	6 (18.2)		1 (3.0)		0	0
Rash	4 (12.1)		0		1 (7.1)	0
Pneumonitis	1 (3.0)		0		0	0
Oral mucositis	5 (15.2)		0		2 (14.3)	0
Hypothyroidism	1 (3.0)		0		0	0
Gum bleeding	2 (6.1)		0		1 (7.1)	0
**Hematologic**						
Decreased neutrophil count	22 (66.7)		3 (9.1)		0	0
Decreased white blood count	23 (69.7)		3 (9.1)		0	0
Anemia	4 (12.1)		0		0	0
Decreased platelet	2 (6.1)		0		1 (7.1)	0
Increased ALT/AST	6 (18.2)		1 (3.0)		1 (7.1)	0
Hypertriglyceridemia	6 (18.2)		0		3 (21.4)	0
Hypercholesterolemia	5 (15.2)		0		2 (14.3)	0
LDL elevation	2 (6.1)		0		0	0
TSH elevation	9 (27.3)		0		3 (21.4)	0

AE, adverse event.

In anlotinib combination therapy group, the incidence of treatment-related hematological and non-hematological AEs was both higher than those of the anlotinib monotherapy group, which is related to the concurrent chemotherapy and immunotherapy. Grade 3-4 AEs were secondary hypertension (n=2,6.1%), hand-foot syndrome (n=2,6.1%), nausea or Vomiting (n=2,6.1%), diarrhea (n=1,3.0%), sensory neurotoxicity (n=1,3.0%), decreased neutrophil count (n=3,9.1%), decreased white blood count (n=3,9.1%) and increased ALT/AST (n=1,3.0%). No patient had an anlotinib dose reduction due to adverse reactions.

## Discussion

Angiogenesis plays a key role in the growth, proliferation and metastasis of a variety of solid tumors. Anti-angiogenic drugs can exert anti-tumor effects by targeting vascular endothelial growth factor (VEGF) and other signal factors, inhibiting their overexpression, and promoting the normalization of tumor blood vessels. As a recombinant humanized monoclonal antibody, which can target and bind with VEGF, reduce neovascularization and inhibit tumor growth, bevacizumab was the first angiogenesis inhibitor approved for clinical use. Currently, bevacizumab has shown promising antitumor activity in a variety of solid tumors, including metastatic colorectal cancer, non-small cell lung cancer (NSCLC), glioblastoma, renal cell carcinoma, ovarian and cervical cancer. However, the clinical value of bevacizumab in metastatic breast cancer remains controversial. E2100, AVADO and RIBBON-1 clinical studies have shown that first-line bevacizumab combined with chemotherapy significantly improved the PFS of metastatic breast cancer patients compared with chemotherapy alone, but did not bring about the benefit of OS (14–16). The AVF2119G, RIBBON-2, and TANIA studies evaluated the value of bevacizumab combined with chemotherapy in the second-line or above therapy of metastatic breast cancer (21–23). These studies demonstrated that adding bevacizumab on the basis of chemotherapy could not improve the PFS and OS of patients.

In addition to bevacizumab, small molecule tyrosine kinase inhibitors (TKIs) can not only act on VEGFR, but also inhibit other related tyrosine kinases, so as to inhibit angiogenesis. Numerous clinical trials confirmed that these TKIs show good efficacy in a variety of solid tumors and become a new anti-angiogenesis strategy (24, 25). Previous studies have explored the efficacy of sunitinib, sorafenib, and apatinib in metastatic breast cancer, and the results suggest that only apatinib shows better efficacy and safety (26, 27). A phase II study evaluated the efficacy and safety of apatinib monotherapy in heavily pretreated metastatic triple negative breast cancer (mTNBC). Median PFS and OS reached 3.3 and 10.6 months, respectively (17). As a novel oral TKI, anlotinib has been approved by the China Food and Drug Administration (CFDA) for advanced non-small cell lung cancer (NSCLC), soft tissue sarcoma and medullary thyroid cancer. Meanwhile, real-world studies have also shown that anlotinib is effective in multiple solid tumors. At present, the clinical efficacy of anlotinib in metastatic breast cancer has rarely been reported. A previous basic study showed that anlotinib inhibits the proliferation and induces apoptosis of MCF‐7 breast cancer cells by downregulating TFAP2C (28). At present, only one phase II clinical study with a small sample size has evaluated the clinical efficacy of anlotinib in metastatic breast cancer (29).

Our present study evaluated the efficacy of anlotinib monotherapy or combination therapy as third-line or above therapy in 47 patients with HER-2 negative metastatic breast cancer. To our knowledge, this is the largest real-world analysis to date investigating the efficacy and safety of anlotinib in breast cancer. The overall ORR and DCR were 21.3% and 74.5%, and median PFS and OS reached 5.0 and 21.0 months. It is worth noting that all the patients enrolled in the present study are heavily pretreated metastatic breast cancer, thirty-eight patients (80.9%) had visceral metastasis, and anlotinib was given as forth-line or above therapy in 59.6% patients. In such a population with a heavy tumor burden, anlotinib has still achieved good clinical efficacy as a late-line treatment manner. Regardless of the NCCN guidelines or the Chinese Society of Clinical Oncology (CSCO) guidelines, there is no standard recommended regimen for the third-line and above treatment of metastatic breast cancer. Therefore, there is no accepted standard treatment regimen to serve as the control group for this study. However, we can review the data of chemotherapy and targeted therapy in the third-line or above treatment of metastatic breast cancer in previous clinical studies. The 304 study is a phase III study, which explored the efficacy of eribulin versus vinorelbine in the treatment of recurrent/metastatic breast cancer. The median PFS in the eribulin-treated group was 2.8 months. The ASCENT study explored the efficacy of sacituzumab govitecan (SG) in the third-line or above treatment of metastatic breast cancer, a median PFS of 4.8 months and median OS of 12.1 months were obtained. Comparing these data, anlotinib showed better clinical efficacy. In TNBC population, the median PFS were 3.5 months and the median OS was 12.6 months, which is consistent with data from previous studies on apatinib in metastatic TNBC (median PFS and OS reached 3.3 and 10.6 months). Baseline clinicopathological characteristics of patients did not affect the clinical efficacy of anlotinib, including metastatic sites type and number of metastatic sites.

At present, the application of anti-angiogenic drugs in breast cancer is mainly in metastatic TNBC patients, and there is little exploration for hormone receptor-positive metastatic breast cancer. Endocrine therapy is an important treatment strategy for patients with HR positive metastatic breast cancer, especially the addition of targeted drugs such as CDK4/6 inhibitors, which significantly improves the prognosis of patients with HR positive metastatic breast cancer (30–32). However, we also face some difficulties in clinical practice. First, CDK4/6 inhibitors are currently not widely used due to drug availability and economic concerns. In the present study, only 4 patients had received CDK4/6 inhibitor treatment. Second, for endocrine resistant breast cancer, novel and feasible treatment strategies still need to be explored. In the present study, 25 HR positive metastatic breast cancer patients received anlotinib therapy. Although there was no statistically significant difference in ORR and DCR between HR-positive and HR-negative groups, the median PFS in HR-positive patients reached 6.5 months, which was significantly better than HR negative group. Our present study confirmed that anlotinib anti-angiogenesis therapy also provide an effective late-line treatment option for HR-positive metastatic breast cancer.

The optimal mode of administration of anlotinib has not been established. Unlike monoclonal antibodies anti-angiogenic drugs, which must be used in combination with chemotherapy or other drugs, small molecule TKIs monotherapy may also have objective clinical efficacy. In the present study, patients who received anlotinib monotherapy obtained no statistically significant ORR and DCR. But patients who received anlotinib combination therapy obtained better PFS than those who received anlotinib monotherapy. Anlotinib combination therapy is the preferred treatment mode, and for patients with poor physical condition, anlotinib monotherapy is also an optional treatment strategy. In anlotinib combination therapy group, except the combination with traditional chemotherapy drugs, some patients received anlotinib in combination with immune checkpoint inhibitors (ICIs) immunotherapy. Antiangiogenic therapy and immunotherapy both act on the tumor microenvironment, and previous preclinical studies have shown that immunotherapy combined with anti-angiogenic drugs synergistically inhibit tumor growth and metastasis (33). Some clinical trials also confirmed the value of antiangiogenic therapy in combination with immunotherapy (34, 35). However, the application of anlotinib combined with ICIs in breast cancer has not yet been reported, our present study shows that anlotinib combined with immunotherapy has also achieved good clinical efficacy in metastatic breast cancer. We also compared the efficacy of different chemotherapy and immunotherapy medications, and there were no significant differences among anlotinib plus capecitabine, nab-paclitaxel or pembrolizumab.

Of the patients enrolled in this study, 14 patients had received prior antiangiogenic therapy, including bevacizumab and apatinib. Whether prior anti-angiogenic therapy will affect the therapeutic efficacy of anlotinib? Our present study showed that the patients who did not received prior anti-angiogenesis therapy had superior DCR (84.8% vs. 50.0%, P=0.012). And simultaneously, although there were no statistically significant differences in ORR, PFS, and OS between the two groups, the absolute values of ORR, PFS, and OS were also better in patients who did not received prior anti-angiogenesis therapy. Therefore, our study suggests that prior antiangiogenic therapy may affect the efficacy of anlotinib.

The most common anti-angiogenic drugs related adverse events are secondary hypertension, hand-foot syndrome, and proteinuria. In previous clinical studies, most of these toxicities can be alleviated by dose adjustment and suspension of administration. In the present study, most of the adverse events in patients received anlotinib therapy were grade 1-2 in severity. The incidence of secondary hypertension, hand-foot syndrome, and proteinuria was consistent with previous relevant clinical trials (36). Antiangiogenic drugs may increase the risk of bleeding. In this study, 3 patients had gum bleeding, but no serious bleeding events such as hemoptysis, gastrointestinal bleeding, and hematuria occurred.

Our present study has some several limitations, because it is an observational and exploratory study and the number of patients enrolled is not very large. The purpose of this study was to explore the clinical value of anti-angiogenesis therapy strategy, and therefore, the results of this study need future validation with large-scale prospective clinical trials. In this study, only a small number of patients received single drug treatment of anlotinib, and more patients received combination therapy. Therefore, this study confirmed the value of anlotinib based scheme as third-line or above therapy for patients with HER-2 negative metastatic breast cancer.

## Conclusion

In conclusion, these data confirm that anlotinib monotherapy or combination therapy provide a viable third-line or above therapeutic strategy in patients with HER-2 negative metastatic breast cancer, a median PFS of 5.0 months was obtained with well tolerated toxicity.

## Data availability statement

The raw data supporting the conclusions of this article will be made available by the authors, without undue reservation.

## Ethics statement

The studies involving human participants were reviewed and approved by The ethics committee of the Henan Provincial People’s Hospital (2021-84). The patients/participants provided their written informed consent to participate in this study.

## Author contributions

All authors contributed to the article and approved the submitted version. YS and HL designed the research, analyzed the data and drafted the paper. YS, ZL, YY and YH were mainly responsible for data collection and analysis. QC, CL, FZ and BN were primarily responsible for statistical analysis.

## Funding

This work was supported by Medical Science and Technique Foundation of Henan Province (No. LHGJ20210055) and Beijing Medical Award Foundation Project (No.YXJL-2020-0941-0748).

## Conflict of interest

The authors declare that the research was conducted in the absence of any commercial or financial relationships that could be construed as a potential conflict of interest.

## Publisher’s note

All claims expressed in this article are solely those of the authors and do not necessarily represent those of their affiliated organizations, or those of the publisher, the editors and the reviewers. Any product that may be evaluated in this article, or claim that may be made by its manufacturer, is not guaranteed or endorsed by the publisher.
