# Supplementary material for: A real-world study of anlotinib as third-line or above therapy in patients with her-2 negative metastatic breast cancer
Source: Front Oncol. 2022 Jul 28;12:939343. doi: 10.3389/fonc.2022.939343 (PMC9366600; doi:10.3389/fonc.2022.939343)
Supplement: Supplementary file 1 [file Table_1.docx]

**Supplementary table 1 Efficacy of anlotinib treatment in patients with Her-2 negative metastatic breast cancer**

| **Parameter** | **Best response** | | | | **ORR** | ***P*** | **DCR** | ***P*** | **Median PFS (95%CI)** | ***P*** | **Median OS (95%CI)** | ***P*** |
| --- | --- | --- | --- | --- | --- | --- | --- | --- | --- | --- | --- | --- |
|  | CR | PR | SD | PD |  |  |  |  |  |  |  |  |
| **Metastatic sites type** |  |  |  |  |  | 0.326 |  | 0.270 |  | 0.625 |  | 0.800 |
| Visceral | 0 | 7 | 20 | 11 | 18.4(7/38) |  | 71.1(27/38) |  | 5.0(3.2-6.8) |  | 21.0(14.2-27.8) |  |
| Non-Visceral | 0 | 3 | 5 | 1 | 33.3(3/9) |  | 88.9(8/9) |  | 5.0(2.1-7.9) |  | 21.5(5.6-37.4) |  |
| **Number of metastatic sites** |  |  |  |  |  | 0.391 |  | 0.074 |  | 0.143 |  | 0.037 |
| 1-2 | 0 | 5 | 11 | 2 | 27.8(5/18) |  | 88.9(16/18) |  | 5.0(2.8-7.2) |  | NR |  |
| ≥ 3 | 0 | 5 | 14 | 10 | 17.2(5/29) |  | 65.5(19/29) |  | 3.0(0.6-5.4) |  | 19.8(10.2-29.4) |  |
| **Prior chemotherapy after metastasis** |  |  |  |  |  | 0.651 |  | 0.961 |  | 0.723 |  | 0.702 |
| With Taxanes | 0 | 8 | 18 | 9 | 22.9(8/35) |  | 74.3(26/35) |  | 5.0(4.0-6.0) |  | 21.5(13.7-29.3) |  |
| Without Taxanes | 0 | 2 | 7 | 3 | 16.7(2/12) |  | 75.0(9/12) |  | 3.0(0-6.2) |  | 16.6(11.1-22.1) |  |
| **Treatment type in combination group** |  |  |  |  |  | 0.861 |  | 0.796 |  | 0.574 |  | 0.582 |
| Anlotinib + capecitabine | 0 | 2 | 6 | 3 | 18.2(2/11) |  | 72.7(8/11) |  | 5.0(2.8-7.2) |  | 15.7(5.9-25.5) |  |
| Anlotinib + nab-paclitaxel | 0 | 3 | 9 | 3 | 20.0(3/15) |  | 80.0(12/15) |  | 5.5(2.7-8.3) |  | 21.0(7.4-34.6) |  |
| Anlotinib + pembrolizumab | 0 | 2 | 4 | 1 | 28.6(2/7) |  | 85.7(6/7) |  | 6.6(2.5-10.7) |  | 22.2(NE) |  |

**Abbreviations:** CR, complete response; PR, partial response; SD, stable disease; PD, progressive disease; ORR, overall response rate; DCR, disease control rate; PFS, progression free survival; OS, overall survival.
